# The Effect of Exogenous Lysozyme Supplementation on Growth Performance, Caecal Fermentation and Microbiota, and Blood Constituents in Growing Rabbits

**DOI:** 10.3390/ani12070899

**Published:** 2022-03-31

**Authors:** Salma H. Abu Hafsa, Amr E. M. Mahmoud, Amal M. A. Fayed, Abdel-Azeem S. Abdel-Azeem

**Affiliations:** 1 Livestock Research Department, Arid Lands Cultivation Research Institute, City of Scientific Research and Technological Applications, New Borg El-Arab, Alexandria 21934, Egypt; 2 Biochemistry Department, Faculty of Agriculture, Fayoum University, Fayoum 63514, Egypt; aem01@fayoum.edu.eg; 3 Animal Production Research Institute, Agricultural Research Center, Giza 12619, Egypt; amalfayed70@yahoo.com; 4 Poultry Production Department, Faculty of Agriculture, Fayoum University, Fayoum 63514, Egypt; asa10@fayoum.edu.eg

**Keywords:** growth performance, nutrient digestibility, volatile fatty acids, caecal microbiota, antioxidant status, rabbit

## Abstract

**Simple Summary:**

Rabbit farming is increasingly requiring non-antibiotic alternatives to improve rabbit gut health to maintain high feeding efficiency and excellent production index. Thus, using a friendly alternative is an appropriate way to protect rabbits from pathogens while also enhancing their performance and welfare. Lysozyme, an enzyme derived from avian egg white, aids in nutrient digestion and absorption and provides protection against bacterial diseases while lowering pollutants’ excretion, such as ammonia. Thus, the aim of this study was to evaluate the effect of lysozyme supplementation in rabbits’ diets on growth performance, caecal fermentation, bacteria population, and blood constituents. The results demonstrated an improvement in rabbit performance, caecal fermentation, blood lipid profile, and antioxidant status due to an increase in beneficial bacteria in lysozyme-treated rabbits. Therefore, supplementing the rabbit diet with lysozyme up to 150 mg/kg is recommended.

**Abstract:**

The effects of exogenous lysozyme supplementation (LYZ) on growth performance, caecal fermentation and microbiota, and blood characteristics were investigated in growing rabbits. A total of 420 growing male V-Line rabbits (30 d old; weighing 528 ± 16 g) were randomly divided into four groups of 105 rabbits each, and monitored for 42 days. Experimental groups included a control group (LYZ0) fed a basal diet without LYZ supplementation, and three treated groups fed the same basal diet supplemented with LYZ at 50, 100, and 150 mg/kg diet, respectively. The results showed a quadratic improvement in the final body weight, daily growth rate, FCR, and digestibility of DM, while the digestibility of OM, CP, EE, NDF, and ADF improved linearly when LYZ supplementation was increased. The dressing percentage increased quadratically when LYZ levels were increased in the rabbit diets. In rabbits fed LYZ diets, *L. acidophilus* counts increased linearly (*p* < 0.05) and *L. cellobiosus*, and *Enterococcus* sp. counts increased quadratically, whereas *E. coli* counts decreased. In the LYZ-supplemented groups, the caecal pH value and NH_3_-N concentration declined quadratically, whereas total VFA, acetic, and butyric acids increased. Total lipids decreased linearly, whilst triglycerides and cholesterol decreased quadratically with LYZ supplementation. Total antioxidant capacity, superoxide dismutase, glutathione S-transferase, and catalase increased quadratically, while malondialdehyde decreased linearly in the LYZ-supplemented groups. In conclusion, exogenous lysozyme administration improved rabbit growth performance and antioxidant status while lowering the blood lipid profile, altering the bacterial population, and regulating caecal fermentation. Therefore, LYZ up to 150 mg/kg can be used as a potential supplement in rabbit feed.

## 1. Introduction

Rabbit farming is gaining popularity as an alternative agricultural enterprise that can be adopted in both rural and urban areas due to rabbits’ small body size, rapid growth rate, short gestation interval, high prolificacy, and ability to utilise forages and by-products as major dietary ingredients [1]. Rabbit meat has a high commercial value and is a popular, nutritious, and a healthful protein source [2]. Rabbits, on the other hand, are susceptible to pathogenic microorganisms, especially when raised in stressful conditions [3]. In the rabbit sector, there is an increasing interest in using natural feed additives for consumer safety [4]. A variety of nutritional treatments, including commercial amino acid and enzyme supplementation, have been employed to promote nutrient utilisation while maintaining economic efficiency with low-protein diets [5]. Exogenous enzymes have been introduced into the animal’s feed as natural alternative products to complement the endogenous enzymatic capability, which increases feed nutritional value [6], and can promote caecal fermentation and vary concentrations of volatile fatty acid, affecting the colonization of beneficial bacteria in the cecum, thus contributing to the maintenance of the animal’s good health. It is common practice to add enzyme preparations to conventional diets to improve feed digestibility, efficiency, and performance in animals [7,8]. Lysozyme extracted from avian egg whites has been applied in rabbit and broiler production as an effective natural growth promoter and antibacterial agent [9,10,11], and it plays an important defense role in the innate immune system in most mammals [12]. Lysozyme is a 1,4-β-N-acetylmuramidase with antimicrobial effects due to its ability to break down the peptidoglycan found in bacterial cell walls, which results in the loss of cellular membrane integrity, causing cell death [13]. Lysozyme is one of the most promising techniques for enhancing rabbit and broiler health and growth by increasing the diversity of gut microbial balance and antioxidative responses [11,14], making it an excellent candidate to replace antibiotics in the animal production industry [15]. Treatment with lysozyme created an abundance of beneficial bacteria and decreased harmful populations in animal guts [10,16,17,18]. Dietary lysozyme also increased rabbit growth rate, blood health, and antibacterial capacity [10,19]. Furthermore, weaned pigs given dietary lysozyme had better growth performance, diversity of beneficial gut microbiota, health of intestinal barriers, and immunological response [12,16,17]. Based on the aforementioned studies and related information, it can be hypothesized that lysozyme would improve the performance, caecal fermentation and microbiota, and antioxidant status of growing rabbits. Therefore, this study was carried out to evaluate the growth performance, carcass characteristics, blood constituents, caecal fermentation, and microbial population of growing rabbits fed lysozyme-supplemented diet.

## 2. Materials and Methods

This study was conducted at “Baldi Farm”, a private commercial farm in Intelligence Land, Fayoum governorate, Egypt, and was approved and permitted by the Institutional Animal Care and Use Committees (Protocol No. 32-2A-0621) of City of Scientific Research and Technological Applications, Alexandria, Egypt.

### 2.1. Experimental Design, Animals, and Diets

A total of 420 growing male V-Line rabbits, 30 d old and weighing 528 ± 16 g, were randomly distributed into four groups of 105 rabbits in a completely randomised experimental design. The control group was fed a basal diet without exogenous LYZ supplementation (LYZ0, control), while the other three groups (LYZ50, LYZ100, and LYZ150) received LYZ supplementation at 50, 100, and 150 mg/1 kg diet, respectively, from weaning to slaughtering (30 to 72 days of age). Exogenous LYZ supplementation was a product with 10% lysozyme and enzyme activity of 500,000 IU/g (Zhejiang Aegis Biotech Co., Ltd., Zhejiang, China). The ingredients of the experimental diets were combined with lysozyme in the presence of molasses before being pelleted to avoid the loss of the additive concentration and biological function. Rabbits were housed individually in double flat galvanised wire batteries (50 cm L × 40 cm H× 35 cm W), in a well-ventilated building and maintained under similar hygienic and environmental conditions, with an ambient temperature of 23 ± 2 °C, 55–65% humidity, and a photoperiod of 16 h light: 8 h dark. Rabbits were fed a pelleted diet to meet their requirements, as according to de Blas and Mateos [20]. The composition of the experimental diet is shown in Table 1. No antibiotics or coccidiostats were supplemented during the experimental period. All cages were equipped with feeding hoppers and drinking nipples, and feed and fresh water were available ad libitum.

### 2.2. Sampling and Measurements

#### 2.2.1. Performance Measurements

The daily growth rate (DGR) was calculated as DGR = (the final live body weight (g)—the initial body weight (g)/days). Final body weight (FBW) was recorded weekly, but feed intake (FI) was recorded daily as follows: FI = feed offered (g) − feed left (g). Feed conversion ratio (FCR; g feed/g gain) was calculated. The mortality rate was recorded daily, and the percentage was recorded for each group at the end of the experiment.

#### 2.2.2. Nutrient Digestibility

A digestibility trial was performed from 60 d to 72 days of age to determine the nutrient digestibility coefficients as according to Perez [21]. Twelve rabbits from each treatment group were housed individually in metabolic cages and fed the experimental rations for 7 days (preliminary period), for adaptation. Then faeces were collected every 24 h prior to feeding in the morning for 5 consecutive days (collection period). Daily faecal samples were taken from each rabbit, oven-dried at 70 °C for 48 h, then ground and stored for proximate chemical analyses. Samples of feed and dried faeces were analysed for dry matter (DM), organic matter (OM), crude protein (CP), crude fiber (CF), neutral detergent fiber (NDF), and acid detergent fiber (ADF). AOAC [22] procedures were used to determine the CP (Method No. 954.01) and ash (Method No. 942.05) contents. The EE was determined as according to the Soxhlet extract method using petroleum ether as an extracting agent (40–60 °C) (Method No. 930.09) [22]. The contents of NDF and ADF were determined using a Tecator Fibretic System, according to the method described by Van Soest [23].

#### 2.2.3. Carcass Traits

At the end of the experiment, twelve rabbits from each group were randomly selected and starved for 12 h with the provision of water ad libitum. Slaughter weight was recorded immediately before slaughter. After slaughtering and bleeding was completed, carcass traits were evaluated and the weights of the heart, liver, spleen, lung, kidneys, and total fat were recorded. The dressing percentage was calculated as carcass weight to slaughter weight ratio.

#### 2.2.4. Caecal Microbiota and Fermentation Patterns

At the end of the experiment (72 days of age), an additional twelve rabbits were taken from each group to measure caecal weight and length, caecal fermentation, and the count of bacteria. After the rabbits were slaughtered, the caecum was carefully excised and emptied with gentle pressure to remove any digesta remaining in the caecum, to determine its weight and length. Caecum length (cm) and full and empty weights (g/g body weight) were calculated. Caecal content samples were transferred to buffered peptone water (Oxoid, Basingstoke, UK), which was immediately used to enumerate the caecal bacteria. Ten-fold dilutions of each sample were performed with buffered peptone water and directly inoculated on de Man–Rogosa–Sharpe (MRS) agar for *Lactobacillus acidophilus*, *Lactobacillus cellobiosus*, and *Enterococcus* sp. Counting, and incubated anaerobically at 37 °C using gas generating kits (Oxoid) for 48 h. *E. coli* were subcultured on MacConkey agar and incubated aerobically at 37 °C for 24 h. Bacterial colonies were counted on plates using an optical colony counter (Gallenkamp, UK), and the overall population was expressed as log cfu/g. Immediately thereafter, caecal contents were strained through two layers of sterile gauze and the resultant strained liquors were used for measuring pH values by an electronic digital pH meter (GLP 21 model; CRISON, Barcelona, Spain). The contents were then centrifuged at 7000× *g* for 10 min at 20 °C. The supernatant fluid was divided into two parts. One part was treated with a solution of 5% orthophosphoric acid (*v*/*v*) plus 1% mercuric chloride (*w*/*v*) (0.1 mL-ml^−1^ sample) for determination of total VFA concentrations and individual VFA proportions, while the other was acidified with 0.2 M hydrochloric acid solution (1 mL-ml^−1^ sample), to be used for determination of ammonia nitrogen (NH_3_-N) concentration. Total VFA concentrations were measured by steam distillation according to Eadie [24]. The concentration of VFA was analysed using High Performance Liquid Chromatography (HPLC; Model Water 600; UV detector, Millipore Crop. Molsheim, France) according to the method of Mathew et al. [25]. After the results of the concentration of the particular VFA had been received, the concentrations (mmoll^−1^) of acetic, propionic, and butyric acids were calculated. The concentration of NH_3_-N in the caecum was determined by using spectrophotometry, as described by Chaney and Marbach [26].

#### 2.2.5. Blood Sampling, Biochemistry and Antioxidant Status

At the end of the experiment, blood samples were collected from 12 slaughtered rabbits per treatment into clean tubes and centrifuged at 3000× *g* for 15 min. Sera were separated and stored at −20 °C until total lipids (TL), triglycerides (TG), total cholesterol (TC), low-density lipoproteins (LDL), high-density lipoproteins (HDL), very low-density lipoproteins (vLDLs), and phospholipids (PL) were determined. Antioxidant enzymes in serum were also measured, including total antioxidant capacity (TAC), superoxide dismutase (SOD), and glutathione S-transferase activity (GST). Blood biochemistry and antioxidant enzymes were determined calorimetrically using standard kits (Biodiagnostic, Cairo, Egypt) and according to the manufacturer’s instructions.

#### 2.2.6. Statistical Analysis

Data were subjected to statistical analyses in a randomized complete block design using general linear model procedures of SAS/STAT (Statistical Analysis System, version 15.1, SAS Institute Inc., Cary, NC, USA) with cage as the experimental unit [27]. Linear and quadratic polynomial contrasts were performed to determine the effect of lysozyme supplementation in the diet. Statistical significance was considered when the *p*-value was less than 0.05. Data obtained were tested by analysis of variance with a one-way design to test the treatment at each sampling, according to the following model:

Y_ij_ = μ + T_i_ + ε_ij_

where y_ij_ denotes the measured value, μ is the overall mean effect, T_i_ is the ith treatment effect, and ε_ij_ denotes the random error associated with the jth rabbits allocated to the ith treatment. The mortality rate was statistically analyzed using a binomial distribution, and the link function was the logit transformation, in (μ/1−μ), where μ was the corresponding rate’s mean value. Results are presented as least-squares means.

## 3. Results

### 3.1. Growth Performance

The effect of LYZ supplementation on growth performance is summarised in Table 2. The initial body weight of rabbits did not differ among treatment groups. Quadratic increases in the FBW (*p* = 0.021) and DGR (*p* = 0.001) were observed when LYZ supplementation were increased in rabbit diets. Rabbits in the LYZ50 and LYZ100 groups had linearly lower FI (*p* = 0.012) than those in other groups, whereas there were no significant differences in FI between the LYZ50 and LYZ100 groups, or between the LYZ0 and LYZ150 groups. In addition, supplementing rabbit diets with LYZ resulted in a linear improvement in FCR (*p* = 0.015). During the trial, ten rabbits died (three for group LYZ0, two for group LYZ50, three for group LYZ100 and two for group LYZ150), and no significant differences were detected in the mortality rate".

### 3.2. Nutrient Digestibility

The digestibility coefficient of DM improved quadratically (*p* = 0.025) in rabbits fed LYZ-supplemented diets. However, with LYZ dietary supplementation, the digestibility coefficients of OM (*p* = 0.01), CP (*p* = 0.011), EE (*p* = 0.020), NDF (*p* = 0.023) and ADF (*p* = 0.012) all improved linearly (Table 3). OM and CP digestibility reached the highest value in the LYZ100 group. There was a linear increase in the digestibility coefficient of CF (*p* = 0.003) in the LYZ100 and LYZ150 groups compared to the other groups, but there were no significant differences in the digestibility coefficients of CF between the LYZ0 and LYZ50 groups or between the LYZ100 and LYZ150 groups. The LYZ150 group exhibited the highest NDF digestibility.

### 3.3. Carcass Traits

The results of the carcass traits are shown in Table 4. The slaughter weight, as well as the proportion of heart, liver, spleen, lung, kidneys, and total fat were not significantly different among the treatment groups. The dressing percentage exhibited a quadratic increase in rabbits fed diets supplemented with LYZ.

### 3.4. Caecal Microbiota and Fermentation Patterns

The effect of supplementation of LYZ on the caecal microbiota and fermentation profile is shown in Table 5. The LYZ150 group had the longest quadratic caecum length and the heaviest empty caecum weight, followed by the LYZ100 and LYZ50 groups, although full caecum weight increased linearly compared with the control group. The count of *L. acidophilus* increased linearly (*p* < 0.05) in rabbits fed LYZ diets, whereas the count of *L. cellobiosus*, and *Enterococcus* sp. increased quadratically. However, the count of *E. coli* showed a quadratic decrease (*p* < 0.05) with the supplementation of LYZ to the rabbit diets. The caecum pH, NH_3_-N concentration, total and proportions of volatile fatty acids were changed when lysozyme was added to the diet. The LYZ supplementation caused a quadratic decrease (*p* < 0.05) in the pH and NH_3_-N concentration. The LYZ100 and LYZ150 groups were similar and had the lowest pH values, followed by the LYZ50 group compared to the control group. Total VFA, as well as butyric and acetic acids proportions increased with LYZ supplementation. Total VFA and butyric proportion reached the highest values in the LYZ100 group, followed by the LYZ150 group. Acetic acid levels increased dramatically in response to increased LYZ levels in rabbit diets, with the LYZ150 group having the highest proportion of acetic acid.

### 3.5. Blood Biochemistry and Antioxidant Status

The effect of different LYZ doses on blood biochemistry and antioxidant status in growing rabbits is shown in Table 6. Total lipids (*p* = 0.03), the LDL: TC ratio (*p* = 0.05) and LDL: HDL ratio (*p* = 0.05) decreased linearly, whereas TG (*p* = 0.03), TC (*p* = 0.02), LDL (*p* = 0.01), vLDL (*p* = 0.03) decreased quadratically with LYZ supplementation. However, LYZ supplementation showed quadratic increases in PL (*p* = 0.05) and HDL (*p* = 0.03). LYZ supplementation resulted in linear increases in HDL: TC ratio (*p* = 0.05) and HDL: LDL ratio (*p* = 0.05). Quadratic increases in serum TAC (*p* = 0.01), SOD (*p* = 0.01), GST (*p* = 0.01), and CAT (*p* = 0.01) due to LYZ supplementation were observed compared to the control group. The LYZ100 and LYZ150 groups had the highest levels of TAC (*p* = 0.01), SOD (*p* = 0.01), GST (*p* = 0.01), and CAT (*p* = 0.01). The LYZ groups had a linear decrease in MDA (*p* = 0.03) compared to the control group, with the LYZ150 group having the lowest level.

## 4. Discussion

A healthy gut is necessary to maintain high feed efficiency and growth performance in farm animals. The present study revealed that the supplementation of lysozyme improved the growth performance, caecal fermentation and bacterial populations, antioxidant status, and overall health status in growing rabbits. Dietary LYZ supplementation enhanced FBW and DGR, with improved FCR. The findings are consistent with those of El-Deep et al. [10], who found that lysozyme at 100–200 mg/kg is efficient for improving growing rabbit performance. Similar studies on rabbits [28,29], poultry [11], and pigs [12,30,31] observed that feeding them lysozyme increased growth performance while improving the gut barrier function and modulated intestinal microbes, resulting in the activation of intestinal immunity with high absorption capacity. The positive effect of LYZ on FBW, DGR, and FCR in this study may be due to the improvement in antioxidants, caecal fermentation, and beneficial microbial abundance in *L. acidophilus*, *L. cellobiosus,* and *Enterococcus* Sp., as well as enhanced nutrient digestibility, which can be attributed to increased nutrient absorption in the rabbit intestine [12,31]. Additionally, the improved growth performance may be attributed to the regulation of metabolism and modulation of the immune response by beneficial microorganisms [32]. Brundig et al. [33] found that dietary LYZ raised 18 known metabolites in the blood that are responsible for protein synthesis through the binding of methionine, threonine, and hydroxyproline, resulting in increased feed utilisation and growth performance. In the present study, the improvement in FCR and growth performance could be explained by the richness of LYZ with amino acids as well as its antioxidant and antibacterial properties, which could increase bacterial activity and feeding efficiency in rabbits [10]. The lysozyme additive had antimicrobial properties with increased protection against harmful microorganisms in the gut of animals [15,34]. Indeed, a high concentration of beneficial bacteria improved feed digestion and nutrient absorption [35].

The size of the caecum and the parameters of caecal fermentation were within the ranges described by Garcia et al. [36]. Caecal size, N-ammonia, and the proportion of butyric and acetic acids were all different among the LYZ-treated and control groups. The VFA produced by fermentation and absorbed in the rabbit’s hindgut is an important source of energy, providing up to 30 to 40% of the energy required for survival [37]. High relative abundances of the beneficial microbiota could indicate more active caecum fermentation, which leads to higher butyrate proportion and improved growth performance [38]. The caecum occupies 40% of the whole-tract content size, and the rabbit ecosystem contains a highly active microbiota that plays an important role in their digestive physiology [39,40]. A variety of factors, including the volume and composition of the rabbits’ diet, influence their pH. Changes in organic acid accumulated in the ingesta could be causing the pH fluctuations.

The present study showed that the caecal microbiota of *Lactobacillus acidophilus*, *Lactobacillus cellobiosus*, and *Enterococcus* were significantly increased when LYZ was added to the rabbit diet, while the count of *E. coli* decreased. The increased *Lactobacillus* count generated by LYZ feeding could be related to LYZ’s antimicrobial, antioxidant, and immunomodulatory properties, which acted as a health indicator for the rabbits [10,11]. *Lactobacillus*, a defensive bacteria, provides a variety of health benefits to the host, including improving the digestion of nutrients [41] and promoting the response of the gut-associated immune system [42]. The antibacterial activities of dietary LYZ suppressed the proliferation of pathogenic bacteria such as *E. coli* in the digestive tract, resulting in improved pig health [12,17]. The current findings are consistent with those of El-Deep [10], who found that using LYZ reduced the count of *E. coli* in growing rabbits. Furthermore, lysozyme acts as an antibacterial agent by hydrolyzing the peptidoglycan in pathogenic microbial cell walls [43].

The present results showed that total VFA concentrations were higher in the LYZ groups than in the control group. According to García et al. [36], total VFA concentrations could reach 99.8 mmoll^−1^ depending on the rabbit’s age, physiological health status, and feed ingredients. Fermentation within the caecum appears to have proceeded normally following LYZ administration, as VFA concentrations amounted to 65.86 mmoll^−1^. The concentration of total VFA in the caecum has been used to estimate microbial activity indirectly since total VFA is the principal product of microbial fermentation. The VFA is rapidly absorbed in rabbits’ hindgut and provides a regular supply of energy for herbivorous animals that use bacterial fermentation as part of their digestion [44]. Lowering total VFA would be nutritionally detrimental to the animal because it stimulates colon mucosal growth [45], a protective factor against pathogenic microbiota [46,47]. Acetate dominates in the rabbit’s caecum, followed by butyrate and then propionate [48]. The molar proportion of butyrate exceeded that of propionate in the rabbit VFA profile, which is in contrast to most herbivorous and omnivorous mammals, which produce more propionate than butyrate in their digestive tracts [49]. The present study revealed that production of acetic and butyric acids increased with LYZ supplementation in the diet at the expense of propionic acid. In contrast, the increased acetogenesis associated with LYZ supplementation may result in the higher production of acetates, at the expense of propionates. Reduced acetogenesis (microbial synthesis of acetate from CO_2_ and H_2_) characterises rabbit caecal fermentation, which is gradually replaced by methanogenesis with age [50]. Acetates contribute to lipogenesis and cholesterologenesis and activate gluconeogenesis from lactate and pyruvate [51]. Lactate is produced by bacterial fermentation in the caecotroph of the stomach, and it is then subsequently consumed during the caecotroph’s digestion in the small intestine [44]. Supplementing with LYZ was also associated with an increase in butyrate, an essential precursor for lipogenesis [51].

Concerning NH_3_-N concentration, LYZ supplementation induced a decremental effect compared to control. According to Macfarlane and Gibson [52], NH_3_-N concentration in the caecum could be influenced by a series of factors, such as H_2_ pressure, chyme reaction, and carbohydrate availability. Proteolytic activity is relatively higher in the rabbit caecum than in ruminants [53], and ammonia concentrations fluctuate between 1.86–23.9 mmoll^−1,^ as reported by Garcia et al. [36]. Additionally, LYZ supplementation reduced the pH of the caecum. When VFA concentration rises and ammonia concentration falls in rabbit caecal chyme, the pH value lowers [36], and so the pH drop associated with LYZ groups coincided well with VFA and NH_3_-N concentrations. Lipid profiles (TL, PL, TG, TC, HDL, LDL, and vLDL), and the LDL: TC ratio, LDL: TC ratio, HDL: LDL ratio, and LDL: HDL ratio in LYZ-fed rabbits were considerably lower than those in the control group, indicating a correlation between lysozyme consumption and lipid profiles. The PL plays an important role in transporting cholesterol and excess TG from the body to the liver, where it is released into the bile juice rather than being deposited on arterial walls or accumulating fat [54]. The decreased TC, TG, TL, and carcass fat deposition in the LYZ-treated rabbits could be explained by the higher PL levels in these rabbits. The capacity of PL to reduce intestinal cholesterol absorption, improve biliary cholesterol excretion, and modify the expression and activity of transcriptional factors and enzymes involved in lipoprotein metabolism could explain these positive effects [55]. HDL is known as “good” cholesterol because it aids in the removal of other, more dangerous types of cholesterol from the circulation. This finding also supports LYZ’s role in rabbits as a health-promoting supplement [55]. Lysozyme reduced fat percentage in carcass components and lowered serum lipid profile compared to the control group, indicating that dietary LYZ had a beneficial effect on lipid-reduction in rabbits.

The TAC is a biomarker for reducing agents in the blood and their ability to scavenge oxidative free radicals [56]. SOD levels were higher in rabbits fed a diet supplemented with lysozyme, indicating that lysozyme improves antioxidant status efficiency. Despite the fact that GST is essential for xenobiotic detoxification within cells, excessive amounts in the blood indicate cell damage [57]. Higher blood total antioxidant activity in LYZ-treated groups may have resulted in improved health as well as higher total antioxidant activity or a reduction in other forms of free radical stress. In both cases, enhanced antioxidant activity reduces oxidative stress exposure. The levels of TAC, SOD, and GST in the serum of rabbits fed dietary LYZ were considerably greater, indicating that these rabbits’ antioxidant defence systems’ ability to scavenge oxidative stress processes had improved. Lysozyme is a good source of antioxidants that can protect cells from free radicals, minimise toxicity, and potentially protect the liver [58]. According to Lin and Yen [59], beneficial gut bacteria create a variety of substances that suppress cytotoxicity, eliminate free radicals, and capture reactive oxygen species. El-Deep et al. [10] and Fritz et al. [60] revealed that lysozyme supplementation enhanced immunity due to changes in immunological and antioxidant status, which is consistent with our findings. Oliver et al. [31] reported that supplementing lysozyme to pig diets resulted in a similar rise in immune responses.

## 5. Conclusions

The results of the present study demonstrated that the dietary supplementation with exogenous dietary lysozyme up to 150 mg/kg can improve growth performance, nutrient digestibility, caecal fermentation, and antioxidant status of growing rabbits.

## Figures and Tables

**Table 1:** Ingredients and chemical composition of the experimental diet (% as dry matter basis).

Feed Ingredients	(%)
Soybean meal (44% CP)	17.5
Wheat bran	15.0
Yellow corn	10.0
Barley	18.0
Alfalfa hay	35.0
Molasses	3.0
DL-Methionine	0.1
Di- Ca- phosphate	0.8
NaCl	0.3
Premix *	0.3
Total	100
Chemical composition (% as dry matter basis):	
Dry matter	90.3
Organic matter	92.1
Nitrogen-free extract	58.6
Crude protein	17.8
Crude fiber	13.4
Ether extract	2.3
Neutral detergent fiber	32.1
Acid detergent fiber	17.1
Digestible energy (MJ/Kg DM)	10.5
Lysine	0.9
Methionine	0.3
Ash	7.9
Calcium	0.9
Phosphors	0.6

* Every 1 kg of vitamins and minerals premix contains (per ton of feed): vitamin D_3_ 450 IU; vitamin A, 6000 IU; vitamin E, 40 mg; vitamin K3, 1 mg; vitamin B_12_, 1200 mg; vitamin B_6_, 39 mg; vitamin B_1_, 3 mg; vitamin B_2_, 1 mg; pantothenic acid, 10 mg; niacin, 180 mg; biotin, 10 mg; folic acid 2.5 mg; manganese, 15 mg; copper, 5 mg; iron, 2.5 mg; zinc, 35 mg; iodine, 0.2 mg; selenium, 0.05 mg; choline chloride, 38 mg.

**Table 2:** Growth performance in growing rabbits fed diet supplemented with different levels of lysozyme.

Parameters	Lysozyme, mg/kg Diet				SEM	*p*-Value	
	LYZ0	LYZ50	LYZ100	LYZ150		Linear	Quadratic
Initial body weight, g	528	528	528	528	24	0.969	0.907
Final body weight, g	1747 ^d^	1832 ^c^	1931 ^b^	2035 ^a^	32	0.003	0.021
Daily growth rate g/d	29.1 ^d^	31.1 ^c^	33.4 ^b^	35.9 ^a^	0.5	<0.001	0.001
Feed intake, g/d	115 ^a^	105 ^b^	105 ^b^	111 ^a^	2	0.002	0.012
Feed conversion ratio	3.95 ^a^	3.3 ^b^	3.13 ^b^	3.10 ^b^	0.27	0.015	0.024
Mortality rate %	2.66	2.00	2.66	2.00	0.73	0.765	0.844

^a–d^ Means within a row with different superscripts are significantly different (*p* < 0.05). Number of observation = 420; 105 rabbits per experimental group.

**Table 3:** Nutrients digestibility in growing rabbits fed diets supplemented with different levels of lysozyme. Digestibility trial performed from 60 to 72 days of age.

Parameters	Lysozyme, mg/kg Diet				SEM	*p*-Value	
	LYZ0	LYZ50	LYZ100	YZ150		Linear	Quadratic
Nutrients digestibility %							
Dry matter	63.4 ^b^	64.8 ^a^	64.8 ^a^	64.9 ^a^	0.11	0.017	0.025
Organic matter	65.1 ^c^	66.9 ^b^	67.8 ^a^	66.9 ^b^	0.89	0.010	0.845
Crude protein	70.9 ^d^	73.8 ^c^	78.5 ^a^	76.1 ^b^	1.46	0.011	1.185
Crude fiber	44.2 ^b^	44.6 ^b^	46.6 ^a^	46.9 ^a^	0.34	0.003	0.335
Ether extract	67.3 ^b^	70.8 ^a^	71.^a^	69.7 ^a^	1.85	0.020	0.097
Neutral detergent fiber	60.5 ^c^	62.5 ^b^	62.1 ^b^	63.0 ^a^	0.27	0.023	0.374
Acid detergent fiber	49.2 ^c^	52.7 ^b^	53.1 ^a^	53.8 ^a^	0.58	0.012	0.528

^a–d^ Means within a row having different superscripts are significantly different (*p* < 0.05). Number of observation = 48; 12 rabbits per experimental group.

**Table 4:** Carcass characteristics in growing rabbits fed diets supplemented with different levels of lysozyme at 72 days of age.

Parameters	Lysozyme, mg/kg Diet				SEM	*p*-Value	
	LYZ0	LYZ50	LYZ100	LYZ150		Linear	Quadratic
Slaughter weight, g	1895	1915	2010	2035	124	0.762	0.733
Dressing percentage, %	54.0 ^d^	56.4 ^c^	57.3 ^b^	58.7 ^a^	0.42	<0.001	0.001
Lungs, %	1.11	1.10	1.20	1.20	0.20	0.743	0.622
Liver, %	3.09	3.14	3.22	3.27	0.83	0.276	0.835
Heart, %	0.31	0.33	0.40	0.34	0.11	0.243	0.352
Spleen, %	0.01	0.12	0.11	0.10	0.01	0.252	0.267
Kidney, %	1.05	1.15	1.09	1.21	0.06	0.185	0.264
Total fat, %	2.39	2.41	2.43	2.38	0.48	0.871	0.365

^a–d^ Means within a row with different superscripts are significantly different (*p* < 0.05). Number of observation = 48; 12 rabbits per experimental group.

**Table 5:** Caecal microbiota and fermentation of growing rabbits fed diets supplemented with different levels of lysozyme at 72 days of age.

Parameters	Lysozyme, mg/kg Diet				SEM	*p*-Value	
	LYZ0	LYZ50	LYZ100	LYZ150		Linear	Quadratic
Caecum length, cm	40.7 ^c^	45.7 ^b^	47.3 ^a^	47.7 ^a^	0.71	0.011	0.013
Full caecum weight, g	95.1 ^c^	114.4 ^b^	115.8 ^b^	119.8 ^a^	1.64	0.011	0.053
Empty caecum weight, g	23.3 ^c^	26.7 ^b^	27.9 ^ab^	28.5 ^a^	1.47	0.005	0.027
Caecal microbial count, log cfu/g caecal digesta							
*Lactobacillus acidophilus*	1.36 ^c^	3.47 ^b^	4.91 ^a^	4.97 ^a^	0.05	0.031	0.054
*Lactobacillus cellobiosus*	1.52 ^c^	1.98 ^b^	2.89 ^a^	2.94 ^a^	0.16	0.016	0.021
*Enterococcus* Sp.	3.57 ^c^	4.21 ^b^	5.75 ^a^	5.95 ^a^	0.19	0.011	0.014
*E. coli*	4.76 ^a^	3.43 ^b^	2.97 ^c^	2.21 ^c^	0.69	<0.001	0.001
Caecal fermentation patterns							
Caecum pH	5.98 ^a^	5.53 ^b^	5.38 ^c^	5.41 ^c^	0.03	<0.001	0.007
NH_3_-N, mmol·L^−1^	13.5 ^a^	12.4 ^b^	12.3 ^b^	12.1 ^b^	0.63	0.002	0.042
TVFA, mmol·L^−1^	55.6 ^d^	62.1 ^c^	65.9 ^a^	63.7 ^b^	0.57	0.016	0.038
Acetic acid, mol/100 mol	44.5 ^c^	48.9 ^b^	49.6 ^ab^	50.1 ^a^	0.55	0.019	0.024
Propionic acid, mol/100 mol	3.34	3.61	3.77	3.32	0.17	0.452	0.898
Butyric acid, mol/100 mol	7.79 ^c^	9.65 ^b^	12.47 ^a^	10.26 ^b^	0.49	0.013	0.052

^a^^–d^ Means within a row with different superscripts are significantly different (*p* < 0.05).TVFA, Total volatile fatty acid. Number of observation = 48; 12 rabbits per experimental group.

**Table 6:** Serum biochemical indices and antioxidant status of growing rabbits fed diets supplemented with different levels of lysozyme.

Parameters	Lysozyme, mg/kg Diet				SEM	*p*-Value	
	LYZ0	LYZ50	LYZ100	LYZ150		Linear	Quadratic
Serum biochemical indices							
TL, mg/dL	354.8 ^a^	348.5 ^b^	349.0 ^b^	341.2 ^b^	3.85	0.03	0.05
PL, mg/dL	138.3 ^b^	141.9 ^a^	146.7 ^a^	144.2 ^a^	2.16	0.05	0.05
TG, mg/dL	71.9 ^a^	63.3 ^b^	58.4 ^bc^	55.2 ^c^	4.52	0.02	0.03
TC, mg/dL	99.2 ^a^	93.9 ^b^	90.2 ^b^	91.9 ^b^	3.06	0.01	0.02
HDL, mg/dL	34.2 ^b^	45.3 ^a^	47.5 ^a^	45.7 ^a^	5.58	0.05	0.03
LDL, mg/dL	50.6 ^a^	35.9 ^b^	30.9 ^c^	35.1 ^b^	5.37	0.05	0.01
vLDL, mg/dL	14.4 ^a^	12.7 ^b^	11.7 ^c^	11.1 ^c^	0.58	0.02	0.03
HDL:TC ratio	0.35 ^b^	0.44 ^a^	0.43 ^a^	0.43 ^a^	0.05	0.05	0.13
LDL:TC ratio	0.51 ^a^	0.38 ^b^	0.34 ^b^	0.38 ^b^	0.14	0.05	0.13
HDL:LDL ratio	0.68 ^c^	1.26 ^b^	1.53 ^a^	1.30 ^b^	0.11	0.05	0.07
LDL:HDL ratio	1.48 ^a^	0.79 ^b^	0.65 ^c^	0.77 ^b^	0.02	0.05	0.07
Antioxidant enzymatic activity							
TAC, mmol/L	2.20 ^c^	2.75 ^b^	3.15 ^a^	3.10 ^a^	0.16	0.001	0.01
SOD, U/L	1.46 ^c^	1.85 ^b^	2.27 ^a^	2.89 ^a^	0.37	0.001	0.01
GST, U/L	116.24 ^c^	136.74 ^b^	156.85 ^a^	153.28 ^a^	6.42	0.001	0.01
CAT, U/L	0.22 ^c^	0.24 ^b^	0.24 ^a^	0.24 ^a^	0.01	0.001	0.01
MDA, μmol/L	2.06 ^a^	1.51 ^b^	1.21 ^bc^	1.11 ^c^	0.68	0.002	0.03

^a^^–c^ Means within a row with different superscripts are significantly different (*p* < 0.05). TL, Total lipids; PL, Phosolipids; TG, Triglyceride; TC, Total cholesterol; HDL, High-density lipoprotein; LDL, Low-density lipoprotein; vLDL, very low-density lipoproteins; TAC, Total antioxidant capacity; SOD, Superoxide dismutase; GST, Glutathione S-transferase; CAT, Catalase; MDA, Malondialdehyde. Number of observation = 48; 12 rabbits per experimental group.

## Data Availability

The data presented in this study are available on request from the corresponding author.
